# Book Reviews

**Published:** 1896-08

**Authors:** 


### Book Reviews.

New Truths in Ophthalmology, as Developed by G. C. Savage, M. D., Professor of Oph thalmology in the Medical Department of the Vanderbilt University, Ex-President Nashville Academy of Medicine, etc. Fifty eight Illustrations. Third Edition. Published by the Author. Printed at the Publishing House of the M. E. Church, South, Nashville, Tenn., 1896.

This work of nearly three hundred pages has undergone revision, and is now in its third edition. The reputation of Dr. Savage, the accomplished editor of the *Ophthalmic Review*, is a guarantee of the merit of his work in the field of ophthalmology.

---

Don'ts For Consumptives, By Charles Wilson Ingraham, M. D., N. Y.

This little work gives to the physician and consumptive practical details of diet, hygiene, and treatment, and shows clearly how life may be prolonged and the prognosis rendered more favorable by attention to the curative forces of nature. The book will doubtless be widely read.

---

Diet for Infants and Children in Health and Disease. By Louis Starr, M. D. Philadelphia: W. B. Saunders, 925 Walnut Street. 1896.

Every doctor has had trouble in making sufficiently clear to the parent or nurse of his little patients, just what proportions of food he wished used and its method of preparation, and therefore Dr. Starr's useful little book on Diet in Infancy and Childhood, will undoubtedly be welcomed by all those engaged in this line of practice.

Printed in detachable sheets so that they can be torn off and left with the mother, are a series of excellently arranged diets such as this author has a well-earned reputation for, the first half being devoted to those in health from the first month onward, while the second half is devoted to diet in the different infantile diseases. The whole completed by pages, also detachable,on the method of preparing different standard diluents and foods.

To any doctor, but perhaps,especially, to the unmarried one who lacks the practical experience one's own children gives one, this book will be valuable, and can be, in the opinion of the writer, unreservedly commended.

---

OBSTETRIC ACCIDENTS, EMERGENCIES AND OPERATIONS. By L. CH. BOISLINIERE, A.M., M.D, LL. D. 8vo. pp, 38. Profusely Illustrated. Philadelphia: W. B. Saunders. 1896. Price $2.00 net.

No one in these days of antiseptic and aseptic surgery can-be considered qualified to write on the subject of Obstetric Emergencies and Operations who is not fully abreast of the very most recent advances in asepsis, or who has not kept up with strides of the modern obstetric art, and no number of years of practice can justify one in writing a book if that book is likely to mislead those beginning their professional life, or those who turn to it as the exponent of the most recent views on the subject. Hence we are unable, despite the fact that there is in this volume much of interest, to recommend this work either to the student, who would find its utter lack of system confusing, or to the practitioner who would not find in it that help he would naturally hope to find.

An author who can to-day recommend swabbing out the uterus in post-partum hemorrhage with pure perchloride of iron and packing it with tampons soaked in the same fluid, one who advises the long-obsolete auton transfusion after hemorrhage and never refers to the now universally used intervenous or sub-mammary saline infusion, one who despite the undoubted trend of modern obstetrics, clings to free bleeding in eclampsia, or recommends as a beginning dose ten minims of dilute hydrocyanic acid—such a writer can only be looked on as a dangerous guide and a blind leader of the blind, and no conscientious reviewer can feel justified in recommending his book to his brother practitioners.

---

*Western Medical Review* is a new and neat double columned monthly edited by Dr. Geo. H. Simmons of Lincoln, Nebraska. We wish it much success.

